# Impression Cytology Is a Non-invasive and Effective Method for Ocular Cell Retrieval of Zika Infected Babies: Perspectives in OMIC Studies

**DOI:** 10.3389/fnmol.2019.00279

**Published:** 2019-12-05

**Authors:** Raquel Hora Barbosa, Maria Luiza B. dos Santos, Thiago P. Silva, Liva Rosa-Fernandes, Ana M. V. Pinto, Pricila S. Spínola, Cibele R. Bonvicino, Priscila V. Fernandes, Evandro Lucena, Giuseppe Palmisano, Rossana C. N. Melo, Claudete Aparecida Araújo Cardoso, Bernardo Lemos

**Affiliations:** ^1^Molecular and Integrative Physiological Sciences Program, Department of Environmental Health, Harvard T.H. Chan School of Public Health, Boston, MA, United States; ^2^Department of Maternal and Child, School of Medicine, Universidade Federal Fluminense, Niterói, Brazil; ^3^Genetics Program, Instituto Nacional de Câncer, Rio de Janeiro, Brazil; ^4^Laboratory of Cellular Biology, Department of Biology, Universidade Federal de Juiz de Fora, Juiz de Fora, Brazil; ^5^Glycoproteomics Laboratory, Department of Parasitology, ICB, Universidade de São Paulo, São Paulo, Brazil; ^6^Biomedical Institute, Universidade Federal Fluminense, Niterói, Brazil; ^7^Genetics Department – Universidade Federal do Rio de Janeiro, Rio de Janeiro, Brazil; ^8^Division of Pathology (DIPAT), Instituto Nacional de Câncer, Rio de Janeiro, Brazil; ^9^Division of Clinical Research, Instituto Nacional de Câncer, Rio de Janeiro, Brazil

**Keywords:** Congenital Zika Syndrome, neurodevelopmental disorders, ocular cells, molecular research, OMIC studies

## Abstract

**Importance:**

Non-invasive techniques for retrieving ocular surface cells from babies infected by zika virus (ZIKV) during the gestational period remain to be determined.

**Objectives:**

The aim of this study was to describe an optimized impression cytology method for the isolation of viable cells from Zika infected babies with and without Congenital Zika Syndrome (CZS) in satisfactory amount and quality to enable easy adoption in the field and application in the context of genomic and molecular approaches.

**Design, Settings, and Participants:**

Ocular surface samples were obtained with a hydrophilic nitrocellulose membrane (through optimized impression cytology method) from twelve babies referred to the Pediatric Service of the Antonio Pedro Hospital, Universidade Federal Fluminense (UFF), Niteroi, Rio de Janeiro, Brazil. After an authorized written informed consent from the parents, samples were collected from both eyes of 12 babies (4 babies with maternal ZIKV exposure during gestation and presence of clinical signs which included ocular abnormalities and microcephaly; 4 babies with maternal ZIKV exposure during gestation but no clinical signs; and 4 unaffected control babies with negative PCR for Zika virus and without clinical signs). Cells were used for microscopy analyses and evaluated for their suitability for downstream molecular applications in transcriptomic and proteomic experiments.

**Results:**

Our optimized impression cytology protocol enabled the capture of a considerable number of viable cells. The microscopic features of the conjunctival epithelial cells were described by both direct analysis of the membrane-attached cells and analysis of cytospinned captured cells using several staining procedures. Epithelial basal, polyhedral and goblet cells were clearly identified in all groups. All cases of ZIKV infected babies showed potential morphological alterations (cell keratinization, pyknosis, karyolysis, anucleation, and vacuolization). Molecular approaches were also performed in parallel. Genomic DNA and RNA were successfully isolated from all samples to enable the establishment of transcriptomic and proteomic studies.

**Conclusions and Relevance:**

Our method proved to be a suitable, fast, and non-invasive tool to obtain ocular cell preparations from babies with and without Zika infection. The method yielded sufficient cells for detailed morphological and molecular analyses of samples. We discuss perspectives for the application of impression cytology in the context of ZIKV studies in basic and clinical research.

## Introduction

Zika virus (ZIKV) is an arbovirus of the *Flavivirus* genus first identified in Uganda – Zika forest, in 1947 (Dick et al., 1952). The virus was detected in the Northeast Region of Brazil in 2015 after an outbreak of cases of acute disease (Cardoso et al., 2015; Zanluca et al., 2015). However, recent studies indicate that the virus circulation in Brazil occurred prior to this epidemic period (Faria et al., 2017; Massad et al., 2017; Metsky et al., 2017; Passos et al., 2017). Congenital Zika Syndrome (CZS) was identified due to the increased incidence of congenital defects associated with ZIKV infection. This led to clinical, epidemiological and experimental studies seeking to address the association between congenital defects and ZIKV infection. Furthermore, the World Health Organization (WHO) recognized ZIKV and associated neurological complications as a long-term public health challenge. A global strategic response plan has been issued to enable detection, prevention, care and support in affected areas (WHO, 2016). Studies were also launched to advance the development of intervention, control, and prevention strategies (Brazilian Ministry of Health, 2018).

Studies on CZS have predominantly involved analysis of brain regions (Mlakar et al., 2016; Oliveira Melo et al., 2016; Driggers et al., 2017; Krauer et al., 2017) but studies of the ocular system have also been conducted. The studies documented characteristic ocular lesions, such as pigment mottling, macular atrophy, chorioretinal atrophy, horizontal nystagmus and optic nerve hypoplasia and atrophy in the context of ZIKV infection (de Paula Freitas et al., 2016; Ventura et al., 2016a, c; Fernandez et al., 2017; Zin et al., 2017; Ventura and Ventura, 2018). Retinal changes occur in about 30–40% of cases and anomalies of the development of the eye may occur in several embryogenesis stages such coloboma, and ocular structure, including eyelid, cornea, iris, zonula, and ciliary body, choroid, retina and optic nerve (Tzelikis et al., 2004; Ventura et al., 2016b). Moreover, anterior ocular alteration associated with prenatal ZIKV infection was observed in CZS newborns (de Paula Freitas et al., 2016, 2017a,b; Yepez et al., 2017). Consequently, screening and long-term monitoring of ocular health are crucial to all children with possible congenital ZIKV infection (Chimelli et al., 2018; Lebov et al., 2018).

Molecular methodologies have been described to investigate the association between ZIKV and neurological impairment using induced pluripotent stem cells and embryonic stem cell lines differentiated in neuroprogenitors, neurons, glial cells and into the brain organoid structures (Chimelli et al., 2018; Lebov et al., 2018). However, the use of non-invasive strategies to study ocular cells in ZIKV-infected babies remains to be established.

Impression cytology of ocular cells is a non-invasive method for external evaluation of ocular lesions (Egbert et al., 1977; Rocha et al., 1999). This technique has been developed since the discovery that cells from the eye outside of the epithelial layer could be removed by filter membrane application to evaluate various conditions of ocular surface impairment (Tseng, 1985). This method has been applied to anatomically locate the conjunctiva, quantify goblet cell density, stage squamous metaplasia staging, differentiate bacterial, viral, allergic, degenerative or tumor affections (Nelson and Wright, 1984; Maskin and Bode, 1986; Paridaens et al., 1992; Nolan et al., 1994; Dart, 1997).

Here we describe, for the first time, a reliable ocular impression cytology protocol for different applications in the field of ZIKV infection. Our method consists of isolation of viable cells in satisfactory number and quality to enable application in the context of genomic and molecular approaches. OMIC technologies are high-throughput methodologies (Boja et al., 2014) that have not yet been coupled with ocular analysis ZIKV infected patients.

As an extension of the CNS, the eye displays similarities to the brain and spinal cord related to anatomy, functionality, response to damage, and immunology (Lleras-Forero and Streit, 2012; London et al., 2013). Moreover, the accessibility of the eye makes it a suitable research tool, enabling the study of processes in the CNS (London et al., 2013). Thus, we propose an optimized and non-invasive alternative for obtaining ocular cells from babies with ocular anomalies caused by ZIKV infection during embryogenesis and its coupling with OMIC applications. Considering the developmental ocular cells precursor origin from neurogenic ectoderm (Lleras-Forero and Streit, 2012; London et al., 2013), the well-documented ZIKV neurotropism with an affinity for neural progenitor cells (Cugola et al., 2016; de Noronha et al., 2016; Mlakar et al., 2016; Tang et al., 2016; van den Pol et al., 2017) and the maternal ZIKV exposure period (weeks of gestation), the methodology presented here may effectively monitor ZIKV impact on genomic and cellular aspects during the life-course.

## Materials and Methods

### Study Design and Ethic Aspects

Twelve babies referred to the Pediatric Service of the Antonio Pedro Hospital, Universidade Federal Fluminense (UFF) were included in this study. All children have been followed by periodical ophthalmological examinations; samples were obtained with an authorized written informed consent from the parents. This study was approved by Universidade Federal Fluminense Ethics Committee and followed the tenets and guidelines of the Declaration of Helsinki.

Ocular surface samples were collected from both eyes of eight babies according to the CZS diagnostic criteria [Patients 1–4 (ZIKV/CZS) = maternal ZIKV exposure during gestation and presence of clinical signs which included ocular abnormalities and microcephaly; Patients 5–8 (asymptomatic ZIKV) = maternal ZIKV exposure during gestation but no clinical signs identified and four unaffected babies: patients 9–12 (control samples/CTRL) = negative PCR, without clinical signs)].

### Impression Cytology and Ocular Surface Cells Capture

**FIGURE 1:**
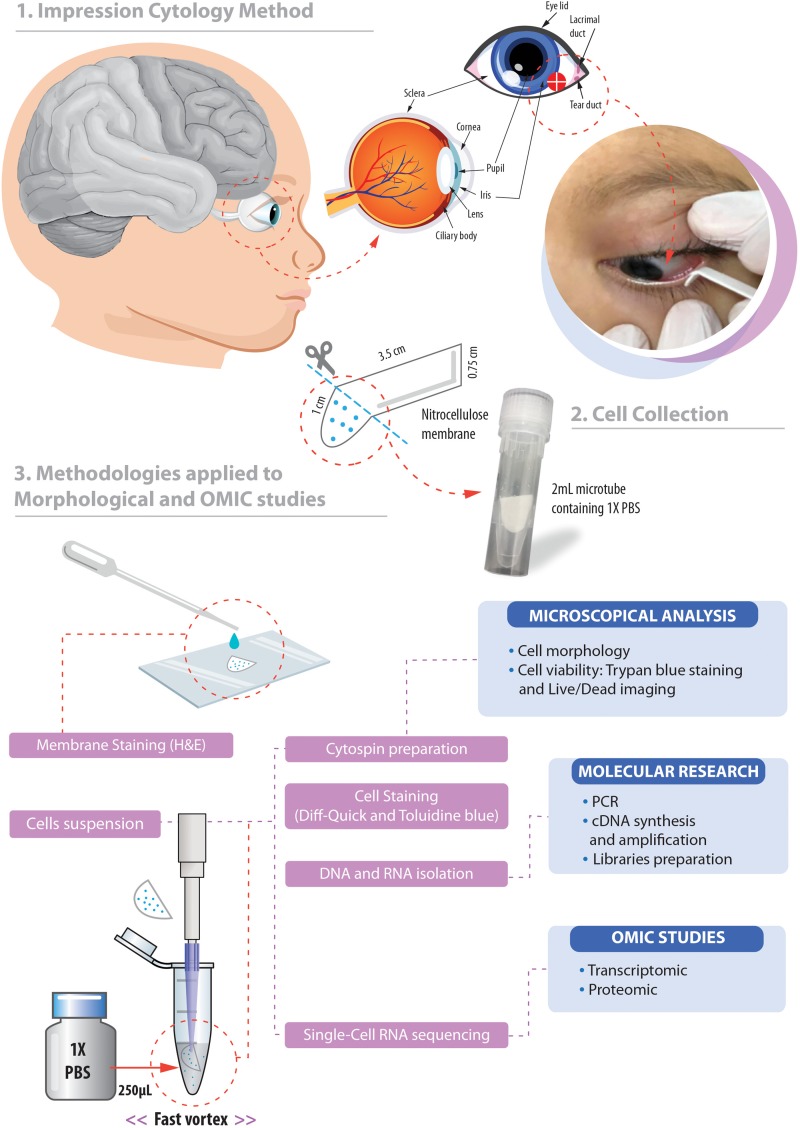
Overview of the non-invasive strategy (impression cytology method) used to collect ocular cells from children with Congenital Zika Syndrome and applied methodologies. **(1)** The eye as an extension of the central nervous system and ocular surface position of the cell collection. **(2)** Impression cytology was optimized (without use of tweezers and pediatric lid speculum) for sample collection. The membrane model has a rounded apex and a long support base that provides a correct positioning and safer method for the collection of baby ocular samples. **(3)** Methodologies applied to different studies of captured cells. Written informed consent was obtained from the parents of this patient for the publication of this image.

A local anesthetic (Proxymetacaine hydrochloride, 0.5% w/v, eye drops, solution) was instilled into the eye before obtaining the ocular surface samples. The samples were collected with a sterilized 0.45 μm, 47 mm white plain hydrophilic nitrocellulose membrane (Millipore Sigma^®^, catalog number HAWP047S0). Each circular membrane was cut into four strips measuring 0.75 cm wide and 4.5 cm long approximately (Figure 1).

The method here described does not use tweezers or pediatric lid speculum for sample collection. A stem of the membrane is used as a collection support. The strip ends were rounded and bent at approximately 1 cm to facilitate printing on the ocular surface and comprises the capture area of ocular cells (Figure 1). The strip end was then pressed on to the inferior bulbar conjunctiva for approximately 5 s. The strip stem was discarded after the collection using sterile scissors (Figure 1).

### Cell Collection and Storage

The cell capture area of the filter membrane was immediately placed in a 1.5 mL tube on ice containing 250 μL 1X PBS (Phosphate Buffered Saline, pH7.4 – Thermo Fisher Scientific, catalog number: 10010023). Tubes containing filter membranes were rapidly vortexed to allow release of adhered cells (Figure 1). Cell suspensions were then used for microscopy analyses or aliquoted in 0.2 mL tubes and stored for additional experiments.

### Cell Analysis and Microscope Image Acquisition

#### Cell Viability

Viability of the collected cells was determined by the trypan blue exclusion test [9 μL of the suspension cells plus 1 μL of 0.4% trypan blue solution (Thermo Fisher Scientific, catalog number: 15250061)]. Viable cells were counted in a Neubauer chamber. Images were acquired using on Axio microscope with 20x/0.35 and 40x/0.55 Zeiss A-Plan objectives (Carl Zeiss, Jena, Germany^1^) and Q-Capture PRO 7 software (Surrey, BC, Canada^2^).

Additionally, the cytospin procedure was used to concentrate cells in suspension on a microscope slide. Cytocentrifuged preparations (200 μL of cell suspension/slide) were obtained in a Cytospin 4 Shandon (Thermo Scientific Corporation, Waltham, MA, United States) at 800 rpm for 5 min at room temperature and stained with LIVE/DEAD^TM^ viability/cytotoxicity kit (Thermo Fisher Scientific, catalog number L3224) as the manufacturer’s instructions. This kit contains a mixture of fluorescent stains (calcein-AM and ethidium homodimer-1) which discriminates live from dead cells by simultaneously staining with green-fluorescent calcein-AM to indicate intracellular esterase activity and red-fluorescent ethidium homodimer-1 to indicate loss of plasma membrane integrity. Analyses were performed on a fluorescence microscope (BX-60, Olympus, Melville, NY, United States) using U-MWB FITC/Texas red filter (488–570 nm excitation wavelengths), which allows simultaneous visualization of both markers.

#### Cell Morphology

To evaluate microscopic features of the captured cells such as morphological types and possible alterations we then used another collection membrane directly stained with hematoxylin and eosin after fixation for 10 min in a fixation solution (100 mL of 70% ethanol, 5 mL of glacial acetic acid, and 5 mL of 37% formaldehyde solution – all solutions are from Merck Millipore). Samples were hydrated with distilled water for 5 min, immersed in Harris’ hematoxylin for 2 min, washed with tap water for 15 min, counterstained with eosin for 30 s, washed with tap water for 5 min, and then dehydrated in 70, 80, 95, and 100% ethyl alcohol (rapid immersion for 5 s each). After these steps, samples were immersed in xylene (10 successive immersions for 5 min each) and mounted on slides cover-slipped using Entellan mounting medium (Merck, Millipore). Images were acquired on Axio microscope using 20x/0.35 and 40x/0.55 Zeiss A-Plan objectives (Carl Zeiss, Jena, Germany, see text footnote 1) and Q-Capture PRO 7 software (Surrey, BC, Canada, see text footnote 2).

Another cytocentrifuged preparation was also stained for morphological analyses. For this, cell suspensions obtained as above were fixed in 4% paraformaldehyde and cytocentrifuged (Cytospin 4 Shandon, Thermo Scientific, 1200 rpm, 10 min). Slides of captured cells (*n* = 3 patients for each group) were prepared in quadruplicate. For each pair of slides, one was stained with a Diff-Quik kit, as the standard procedure, and the other one with 0.5% toluidine blue O solution (Fisher Scientific) for 5 min. Cells were analyzed on a BX-60 Olympus microscope. A total of 423 cells were randomly analyzed (*n* = 103 for control group; *n* = 150 for ZIKV; *n* = 170 for ZIKV/CZS) for a qualitative and quantitative evaluation of morphological alterations. The one-way ANOVA followed by Tukey multiple comparisons test were performed using GraphPad Prism version 6.00 for Windows (GraphPad Software, La Jolla, CA, United States).

#### Molecular Analyses

To evaluate the integrity and quality of the genomic material of captured ocular surface cells, DNA was isolated according to QIAamp DNA mini kit (Qiagen, Valencia, CA, United States), which is indicated for swabs, body fluids or washed cells. Thereafter, we used a pair of primers to amplify a region (exon 4) of the *MECP2* gene that possess a significant role in embryonic development. The forward: 5′- GGA AAG GAC TGA AGA CCT GTA AG - 3′ and Reverse 5′- CTC CCT CCC CTC GGT GTT TG - 3′ (fragment size = 372 pb) primers were used and PCR conditions with modifications were applied according to previous report (Monnerat et al., 2010). The PCR was performed in a total volume of 25 μL, containing 100 ng genomic DNA, 1 × PCR buffer (50 mM KCl, 10 mM Tris–HCl [pH 8.3], 1.5 mM MgCl2), 0.05 mM dNTP, 1.88 pmol of each primer, and 1.25U of the Platinum Taq DNA polymerase (Platinum^TM^ Taq DNA polymerase, Catalog number: 10966026; Thermo Fisher Scientific). Touchdown program was used in the Veriti 96 Applied Biosystems PCR thermal cycler and all samples were denatured at 95°C for 2 min and 30 s, followed by: 25 cycles of 94°C for 30 s, 65°C for 30 s, and 72°C for 1 min 45 s, followed by 10 cycles of 94°C for 30 s, 51°C for 30 s and 72°C for 1 min 30 s then a final extension 72°C for 5 min. For each sample, 5 μL of the final PCR product was checked by 1% agarose gel electrophoresis.

For transcriptomic experiments 9 μL of suspension solution (after fast vortex of capture membrane; Figure 1) containing a maximum number of 150 cells that could be processed for RNAseq (Expert Single Cell Sequencing Services - SingulOmics, 2018; Novogene, 2018) including cDNA synthesis and amplification, library preparation, sequencing (10 million paired-end reads) and data analysis. Here, total RNA quality assessment was performed using the 2100 Bioanalyzer (Agilent, 2018), a microfluidic platform for the electrophoretic separation of biomolecules that is useful for identifying contaminated and degraded RNA (Figures 4, 6). For proteomics experiments proteins can be extracted from the membrane, digested with trypsin and analyzed in a data-dependent manner by nanoflow liquid chromatography coupled to high accuracy and resolution mass spectrometry (Orbitrap Fusion tribrid, Thermo Fisher).

## Results

### Clinical Aspects

Samples were collected from both eyes of eight boys and four girls’ babies with 21 months median age (Table 1). ZIKV infected babies according to the CZS diagnostic criteria (four babies with positive PCR for Zika virus in gestation and presence of clinical signs which included ocular abnormalities and microcephaly – ZIKV infection predominantly in the first trimester), four babies with positive PCR for Zika virus during gestation (occurring in the second and third trimester) but no clinical signs identified and four unaffected babies (control samples/negative PCR, without clinical signs). All babies were absent of external eye disorder at the time of sample collection. Other congenital infections such as toxoplasmosis, rubella, CMV, HSV, syphilis, and HIV were ruled out by serology.

**TABLE 1:** Clinical data.

**Subject’s ID**	**Gender**	**Status (ZIKV exposition during gestation)**	**Status (for microcephaly)**	**Vision impairment**	**Ocular alteration**	**Age at samples collection (in months)**	**Maternal ZIKV infection symptoms and PCR**
#1	M	Exposed	Affected	Yes. Bilaterally	Bilateral optic discs hypoplasia, abnormal ocular pigment deposition and macular atrophy	21	1° trimester (8th week of gestation)
#2	M	Exposed	Affected	Yes. Bilaterally	Increased optic disc excavation, macular pigment mottling and absent foveal reflex	22	1° trimester (9th week of gestation)
#3	M	Exposed	Affected	Yes. Bilaterally	Increased optic disc excavation	19	1° trimester (10th week of gestation)
#4	F	Exposed	Affected	Yes. Bilaterally	Right eye: Optic disc pallor, paramacular atrophy, abnormal ocular pigment deposition and absent foveal reflex; Left Eye: Optic disc pallor and foveal reflex reduction	27	1° trimester (8th week of gestation)
#5	M	Exposed	Non-affected	No	–	21	2° trimester (18th week of gestation)
#6	M	Exposed	Non-affected	No	–	20	2° trimester (16th week of gestation)
#7	F	Exposed	Non-affected	No	–	24	3° trimester (33th week of gestation)
#8	F	Exposed	Non-affected	No	–	24	3° trimester (24th week of gestation)
#9	M	Non-exposed	Non-affected	No	–	9	–
#10	F	Non-exposed	Non-affected	No	–	21	–
#11	M	Non-exposed	Non-affected	No	–	24	–
#12	M	Non-exposed	Non-affected	No	–	19	–

### Cell Visualization, Counting, Distribution, and Morphological Aspects

Here we developed a membrane model for cell collection with a rounded apex and a long base of support that provided a correct positioning for the capture time and for fixing and staining. We used the membrane extremity as collection support not requiring the use of tweezers and pediatric lid speculum (Figure 1). The impression cytology with nitrocellulose membrane model we developed here is effective for ocular surface cell capture. We observed the presence of cells in all collection membranes. In only 9 μL of cell suspension after fast vortex (from an initial total of 250 μL), the number of cells retrieved ranged from 15 to ∼150 cells. Most cells remained attached to the membrane.

**FIGURE 2:**
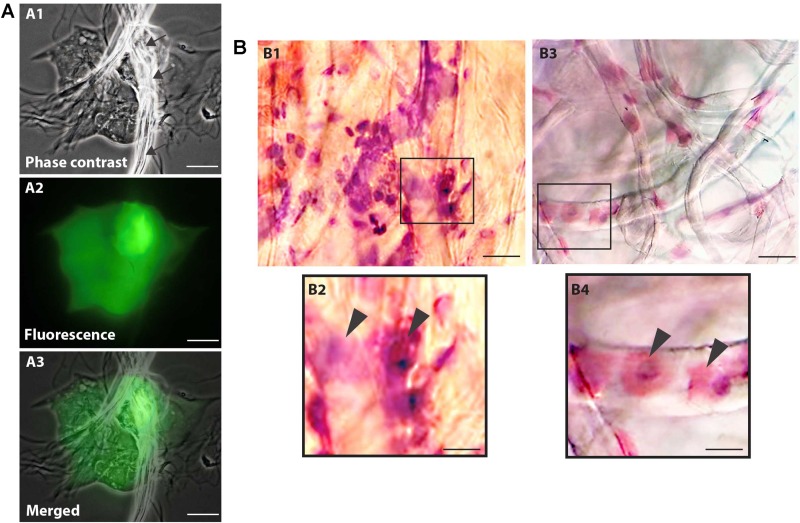
Nitrocellulose membrane fibers impregnated by ocular surface cells from CZS patients **(A,B1,B2)** and uninfected controls **(B3,B4)**. **(A)** Representative live cells are shown by phase contrast **(A1)** and fluorescence microscopy **(A2)**. An overlay of these two images is seen in panel **(A3)**. Note in **(A1)** some fibers from the nitrocellulose membrane on the cells (indicated by arrows). Viable cells fluoresce in bright green after staining with LIVE/DEAD^®^ cell viability/cytotoxicity assay. Cells were imaged 5 h after collection. **(B)** Representative membranes directly stained with hematoxylin/eosin. Membrane-attached cells are indicated by arrowheads in higher magnification in panels **(B2)** and **(B3)**. Scale bars = 20 μm.

Cell viability evaluations by trypan blue test of captured cell suspensions showed that most cells (> 95%) were viable immediately after collection in all groups and different epithelial and goblet cells were observed in Neubauer chamber. We detected the presence of viable cells adhered to the collection membrane (attached to the nitrocellulose fibers) 5 h after application of impression cytology (Figure 2A).

Microscopic analysis of the stained membranes (Figure 2B), showed presence of varying amounts of cells in all of them. Individuals infected with ZIKV (Figures 2B1,B2) showed apparently more cells attached to the membrane when compared with control subjects (Figures 2B3,B4). Some impression areas stained more intensely likely due to multilayering of the cells. The morphologic evaluation was impaired in these regions. Of note, we observed that the eyes with excessive tearing had worse yields in terms of the number of captured cells; however, the differences were not large and this did not affect the viability of the preparations for downstream procedures.

**FIGURE 3:**
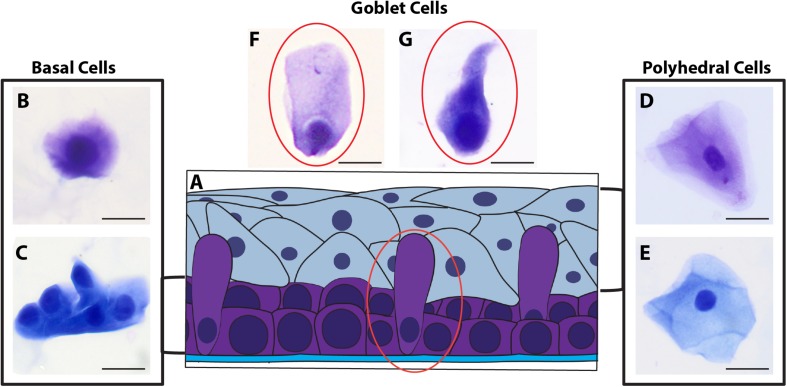
Different ocular surface cells collected from impression cytology. **(A)** Human conjunctiva cell types pattern. Representative basal, polyhedral and goblet cells from uninfected **(B–D, F)** and ZIKV-infected children asymptomatic **(E,G)** show normal morphology. Cytocentrifuge preparations were stained with Diff-Quik **(B,D,F)** or toluidine blue **(C,E,G)**. Scale bars = 20 μm.

**FIGURE 4:**
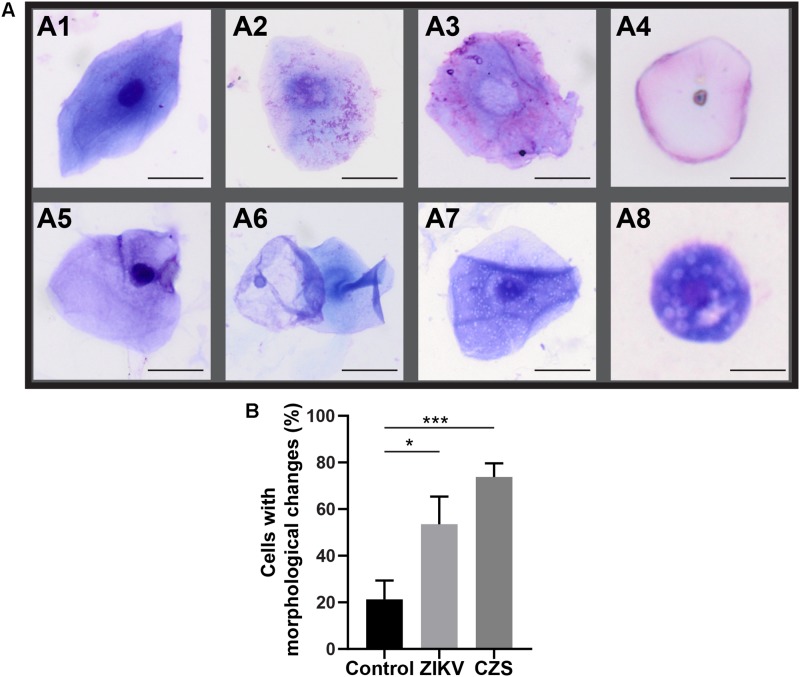
Morphological changes observed in ocular surface cells from ZIKV infected children asymptomatic **(A1,A2)** and CZS children **(A3–A8)**. **(A)** Cell changes included mild to moderate keratinization **(A1–A4)**, pyknosis **(A4,A5)**, karyolysis **(A2,A3)**, anucleation **(A6),** and vacuolization **(A7,A8)**. **(B)** Morphological changes significantly increased in the infected groups. A total of 423 cells was analyzed and quantitated. ^∗^*P* = 0.01, ^∗∗∗^*P* = 0.0009. Cytocentrifuge preparations were stained with Diff-Quik **(A1–A4)** or toluidine blue **(A5–A8)**. Scale bars = 20 μm.

**FIGURE 5:**
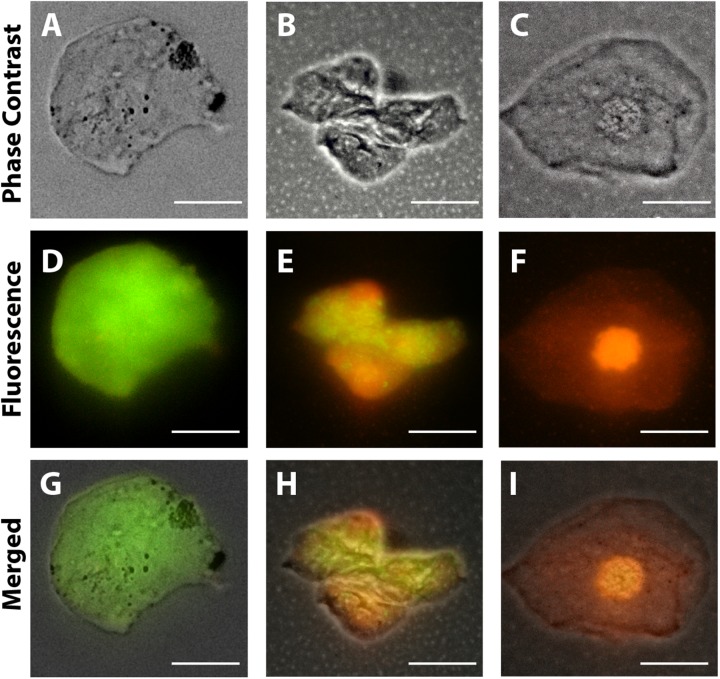
Representative viable and non-viable cells recovered from the conjunctiva of children using an impression cytology method. Live cells fluoresce green whereas dead cells with compromised membranes fluoresce red-orange after staining with LIVE/DEAD cell viability/cytotoxicity assay. Panels **(A,D,G)** are from uninfected patients; panels **(B,E,H)** are from ZIKV patients (positive PCR during gestation and asymptomatic); panels **(C,F,I)** are from CZS children (positive PCR with diagnosed clinical signs: ocular abnormalities and microcephaly). Cells were imaged 5 h after collection. Scale bars = 20 μm.

Morphological cell analyses were performed after cytospinning the cell suspensions which facilitated adhesion of the cells on the slides and resulted in enhanced visualization of cell features (Figure 3). The following cell types, characteristic of the conjunctiva, were identified in all groups: epithelial basal cells, epithelial polyhedral cells and goblet cells (Figure 3A). Epithelial cells of the basal layer were seen individually or in small clusters, with a round to oval shape and a central nucleus and scant cytoplasm (Figures 3B,C). Basal cells stained more strongly compared to other epithelial cells. Intermediate and more superficial epithelial cells were recognized by their polyhedral and abundant cytoplasm with a small and central nucleus (Figures 3D,E). Goblet cells were identified by their typical morphology: an eccentric nucleus and a pale cytoplasm in their apical region (Figures 3F,G). When the groups were compared, morphological changes (Figure 4A) significantly increased in cells collected from ZIKV infected patients, predominantly in those with clinical signs (CZS), compared to uninfected controls (Figure 4B). These included nuclear and cytoplasmic alterations such as mild to moderate keratinization (Figures 4A1–A4), pyknosis (Figures 4A4,A5), karyolysis (Figures 4A2,A3), anucleation (Figure 4A6) and cytoplasmic vacuolization (Figures 4A7,A8). In addition live cells were imaged by intense, uniform green fluorescence while dead cells fluoresced orange-red after staining with a live/dead viability kit (Figure 5).

### Molecular Applications and Genomic Analyses

**FIGURE 6:**
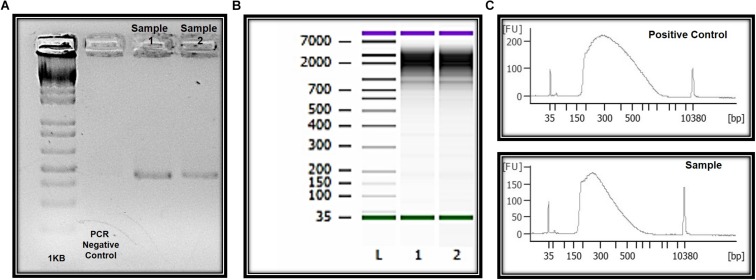
Molecular and “OMIC” research perspectives. **(A)** Electrophoresis in 1.5% agarose gel and the amplification of a 350 bp fragment correspondent to amplicon four of the *MECP2* gene in DNA of the ocular surface cells. **(B)** Quality and integrity RNA analysis. L = ladder, 1 and 2 = CZS children. **(C)** Quality control libraries – comparative concentrations for positive control (of the used method) and healthy child (control sample of this study). Panels **(B**,**C)** were provided by Singulomics©.

Genomic DNA was successfully isolated from all samples. The amount of genomic DNA ranged from 10 ng/μL to as much as 70 ng/μL, with good integrity, and sufficient for successful PCR reactions. A unique fragment of 372 bp correspondent to partial region of exon 4 of the *MECP2* gene was detected in all samples (Figure 6). We also obtained whole and viable cells in good quality for downstream applications using transcriptomic, epigenetic, or proteomic approaches (Figure 6 and unpublished data). As an example, RNA preparations were obtained and proceed with RNA-seq. Given the small number of cells in some preps, we conducted whole transcriptome PCR amplification prior to sequencing (Expert Single Cell Sequencing Services - SingulOmics, 2018; Novogene, 2018). Sufficient RNA and transcriptome libraries were obtained for all samples. Transcriptomic and proteomic analysis revealed differences between the controls and ZIKV exposed groups (*unpublished data*).

## Discussion

Impression cytology has been shown to be a simple and reproducible technique that can be successfully performed in preterm or term infants (Agilent, 2018). However, the methods previously described mostly used tweezers and/or pediatric lid speculum for the collection of samples. Furthermore, some authors have reported difficulties in obtaining adequate samples of infants (Natadisastra et al., 1988; Kjolhede et al., 1989; Hughes et al., 1997). Here we optimize for the first time the technique for use in OMIC studies and discard the use of invasive apparatus, enabling a safer and more effective method for the collection of baby ocular samples. The optimized protocols described here can be straightforwardly performed with sufficient training and easily scaled for analyses of larger clinical populations and under variety clinical settings. The protocol is also ideal for implementation in field conditions.

### Ocular Surface Cells and Applications in Zika Studies

Approximately 50% of children with CZS and microcephaly present serious eye diseases (de Paula Freitas et al., 2016). Furthermore, the ZIKV has the potential to survive for long periods in ocular tissue and potentially cause outcomes that will only be manifested later in life (Bandyopadhyay et al., 2018). Considering the cellular tropism of ZIKV in the eye, ZIKV RNA was detected in all eye regions, included analysis of the anterior (cornea, iris, and lens) and posterior (neurosensory retina, retinal pigment epithelium/choroid complex, and optic nerve) chambers (Miner et al., 2016).

The human ocular surface, a specialized region derived from neurogenic ectoderm which includes the corneal, limbal, and conjunctival stratified epithelia, plays an essential role in ocular system (reviewed in Gokuladhas et al., 2017). Embryological development of the human ocular surface is a complex process, which involves progenitor cellular interactions between various ectodermal eye precursors and mesodermal-derived tissues. Side by side interactions between the neural ectoderm-derived optic vesicles, lens plaque, and facial/cranial ectoderm form the progenitor corneal epithelium and the pluripotential cells of the corneal limbus. Interactions between the early neural tube and the adjacent ectoderm give rise to the neural crest cells, which are a highly migratory and multipotent cell population able to migrate between the lens and presumptive corneal epithelium to form the corneal endothelium, other epithelia of the ocular surface and the stromal keratocytes in a highly dependent process. Innervation of the corneal stroma and epithelium originates from the neural crest- and ectodermal placode-derived trigeminal ganglion (Tripathi and Tripathi, 1990; Barishak, 2001; Swamynathan, 2013; Dhouailly et al., 2014; Lwigale, 2015; Mizoguchi et al., 2015; Forrester et al., 2016).

Due to Zika virus neurotropism to infect neural cells in human embryonic development, the ocular cells obtained in this study may represent an adequate model for the analysis of molecular alterations resulting from ZIKV virus and neuronal cells interaction. Moreover, since viral ZIKV RNA may be present in ocular fluids (in tears and lacrimal glands) (Miner et al., 2016; Swaminathan et al., 2016; Tan et al., 2017), and ZIKV can infect human primary corneal epithelial cells (Singh et al., 2019), in our study we provide for the first time a methodology for cell capture with different perspectives of application. For example, the technique is applicable to the immunolocalization of a wide range of proteins, including detection of ZIKV antigens; to viral analysis through Real Time PCR and ultrastructural microscopy. Studies of the cellular anatomy, physiology and molecular aspects of the ocular surface are essential for understanding ZIKV-associated ocular and neurologic disorders.

Despite their importance, many questions about the genetic characteristics of conjunctival cells (mainly goblet cells) are poorly studied and deserve further exploration (Gipson, 2016). Moreover, the molecular and morphological aspects of human conjunctival stem cells also have not been clearly elucidated (Hughes et al., 1997). Since ZIKV infection has been related to central nervous system abnormalities, the investigation of easily accessible and meaningful tissues is crucial. The *MECP2* gene, for instance, has been linked to Rett syndrome and Angelman syndrome, X-linked

mental retardation, neonatal encephalopathy (severe brain dysfunction in males who live only into early childhood), some cases of autism and systemic lupus erythematosus (Online Mendelian Inheritance in Man [OMIM], 2018). Here, with ocular surface cells, obtained non-invasively, we have successfully standardized a molecular study protocol for the *MECP2* gene and can be optimized for several other molecular studies involving other genes of interest investigating the ZIKV and microcephaly association.

Molecular and cellular events, fundamental to embryological development, postnatal maturation, and maintenance of the ocular surface, are specifically regulated through advanced gene expression mechanisms. Several studies suggest a significant discrepancy between transcription and protein levels in specific cells, indicating that mechanisms related to the regulation of alternative splicing, transcript stability, translation efficiency, protein stability also participate intrinsically in gene expression (Morris et al., 2010; Dahan et al., 2011). With the introduction of transcriptomic and proteomics tools, we can compare the findings between the corresponding transcript and protein levels. In this study, we showed cells to be viable both for transcriptomic research via RNAseq technology and for proteomic validation. The quality of the RNA and libraries obtained in the study of transcriptome profiles is crucial for generating accurate and informative results. Transcriptome and proteomic profiling revealed differences between exposed and controls babies. However, this work focuses objectively on the detailed report of the methods performed to obtain viable cells from infants exposed and not exposed to zika virus with appropriate conditions for the establishment of molecular and OMICs procedures. The large-scale data generated from the different high throughput analyses (transcriptomics and proteomics) are detailed in complementary and separate studies (submitted).

The strategies used here enabled clear detection for the first time of statistically significant morphological changes in cells from ZIKV-infected patients such as cytoplasmic keratinization and nuclear alterations as observed in other ocular disorders using cytology impression approach (Singh et al., 2017). To this date, this is the first study using an approach with perspectives in morphological, molecular and “*OMICs*” research from ocular samples captured by impression cytology of babies with CZS. Studies of mechanisms involved in CZS in ocular cells require rapid, highly reproducible, and accurate quantification and can be successfully achieved with impression cytology. Ocular cell surface capture offers a powerful model for studying the pathways involved in ocular diseases associated with ZIKV.

## Conclusion

The similarity of the eye to other parts of the central nervous system makes it a viable model for studying biological processes in health and CNS pathologies, like Congenital Zika Syndrome. The impression cytology with nitrocellulose membrane model developed and described in this study is a safe and effective method for the collection of ocular surface cells from babies. The sampling technique is easily implemented in field conditions and can be applied to morphological, molecular and “OMIC” analyses of Zika infected patients. On the basis of the evidence described in this study, ocular cell capture has substantial benefits as research tools for central nervous system disorders, include Congenital Zika Syndrome.

## Data Availability Statement

All datasets generated for this study are included in the article/supplementary material.

## Ethics Statement

Study design and ethic aspects: Twelve babies referred to the Pediatric Service of the Antonio Pedro Hospital, Universidade Federal Fluminense (UFF) were included in this study. All children have been followed by periodical ophthalmological examinations; samples were obtained with an authorized written informed consent. This study was approved by the Universidade Federal Fluminense Ethics Committee and followed the tenets and guidelines of the Declaration of Helsinki.

## Author Contributions

RB conceived the original idea, wrote the project study, provided funding, designed the experiments, organized the works groups (cellular, molecular and OMICs), collected the respective informed and written parents’ consents, performed the sample preparation and storage, the analytical method development, data interpretation and wrote the manuscript. MS collected the impression cytology membranes. MS and CC performed the cohort selection. CC coordinates the clinical follow-up study. AP, PS, CB, and PF assisted on analytical method development and revision of the manuscript. LR-F and GP performed the proteomic method development. TS and RM performed the microscopy and morphological analyses. RM and EL assisted on analytical method development and critical revision of the manuscript. BL assisted in the experimental design and revision of the manuscript. All authors contributed in editing the manuscript and approved the final version.

## Conflict of Interest

The authors declare that the research was conducted in the absence of any commercial or financial relationships that could be construed as a potential conflict of interest.

## Footnotes

1 http://www.zeiss.com

2 www.qimaging.com
